# On the Treatment of Femoral Hernia

**Published:** 1896-04-11

**Authors:** W. McAdam Eccles

**Affiliations:** Assistant Surgeon to the City of London Truss Society, and to the West London Hospital, Surgeon to the St. Marylebone Dispensary, &c.


### ON THE TREATMENT OF FEMORAL HERNIA.

(*Continued from page* 414.)

By W. McAdam Eccles, M.S.Lond., F.R.C.S.Eng., Assistant Surgeon to the City of London Truss Society, and to the West London Hospital, Surgeon to the St. Marylebone Dispensary, &c.

### II.—Irreducible Femoral Hernia.

Femoral herniæ, if equal numbers be compared, will be found to be much more liable to become irreducible than inguinal, the proportion being quite ten to one. This fact alone shows the great necessity that there is for care and efficiency in the treatment of femoral ruptures.

The natural disinclination of the female sex to have such a complaint as a hernia seen and treated, through false modesty, is no doubt one of the reasons why these protrusions are prone to become irreducible through neglect. But it must be remembered that the anatomical conditions of a femoral hernia also act in producing irreducibility.

Irreducible femoral herniæ are for the most part small in size, but occasionally they assume larger proportions. Small protrusions when they resist reduction may be treated in one or other of two ways, either by the application of an ordinary femoral truss but with the pad concave, or by operation, when a radical cure may at the same time be aimed at.

The effect of the continuous pressure of a hollow pad upon the protrusion is that in quite half the number of cases in which it is employed reduction is sooner or later obtained. There are certain rules to be followed by patients wearing a hollow pad truss for an irreducible femoral hernia. They must clearly understand that the object of the truss is to secure reduction of the contents of the sac, and it is therefore a truss which is only to be worn until such has been accomplished. Periodical visits, say once in three or four weeks, should be paid to the surgeon, in order that proper taxis may be applied to the swelling. The truss must be regularly kept on at night, a point which is often disobeyed by the patient. There is no need for the patient to assume the horizontal position continuously during the treatment. If the hernia be too large to be satisfactorily covered by the ordinary sized hollow pad, it will be necessary to make a special one for it, and the measurements taken both transversely and vertically for this pad should be full, since the necessary covering of the pad somewhat diminishes its hollow.

In some of these cases of large irreducible femoral hernia it is advisable to employ, in addition to the hollow pad, the thigh belt which was described in the preceding paper, so that the pad may be kept down over the hernial protrusion. Continuous pressure over an enterocele produces no untoward symptoms as it has been thought to do by some. Failure to bring about reduction by pressure is most usually the result of carelessness on the part of the patient. For a hernia to become irreducible is in the majority of cases evidence of some neglect, and to continue so is almost indubitable confirmation of the suspicion in a large number of cases.

If after a careful and extended attempt to procure reduction by pressure the hernia remains irreducible, two courses of treatment are open: Firstly, a continuation of the application of a hollow pad truss, or, secondly, the performance of herniotomy. I think that the former is a bad method in the majority of instances, but must be adhered to in patients who are aged or very feeble, and where there is any serious constitutional disease such as albuminuria, tubercular or cancerous cachexia, diabetes, &c. A hollow pad on an irreducible femoral epiplocele, provided it prevents the descent of gut, may be considered satisfactory, but I cannot recommend its use in irreducible femoral enteroceles or entero-epiploceles. Here, unless there are very distinct counter-indications an operation should be urged, and, if permitted, undertaken.

In operating on a femoral rupture the utmost precautions must be observed in order to secure asepsis. A vertical incision should be made quite over the inner half of the swelling, commencing well above the hernia and continued down to at least half-an-inch below it. Careful dissection will soon expose the sac, which is however, sometimes difficult to recognise. The aborescent vessels on its exterior are a myth, and its shining surface will only be seen on its interior after the sac has been opened. But commonly the sac has a distinctly bluish tinge when thoroughly exposed, and gives a very definite cyst-like feel and outline to the finger, especially if fluid be present and the walls at all tense. In certain circumstances a thin-walled sac may be mistaken for bowel, and may be manipulated so as to produce reduction which necessarily will not occur.

The sac being defined it is to be opened with great care to prevent any injury to the contents in so doing. Adhesions may now be found between the omentum or gut and the inner surface of the sac, or between different parts of the contents themselves. These must be dealt with one by one, and if necessary ligatures must be used, and should consist of fine boiled silk. All adhesions having been separated the gut is to be returned to the abdomen, and any altered omentum tied and cut off, the stump being placed within the peritoneal cavity, and never used as a plug and fastened in the femoral ring. The sac being now quite clear of contents, is thoroughly separated from its surroundings, and its neck transfixed and ligatured with silk, the part beyond the ligature being then cut away. This simple operation, without any further dealing with the femoral ring, is sufficient in some cases, and has been strongly advocated by high authority. Several methods are, however, in vogue by which an attempt is made to close more or less completely the ring itself. The anatomical conditions which obtain in a femoral hernia are such as make closure of the ring a difficult matter, to say the least of it. The proximity of the large common femoral vein, the unyielding character of the tissues involved, and other factors, all combine against its accomplishment. If, however, a strong silk suture, threaded on a long-handled needle, be made to take up the pubic portion of the *fascia lata* and the posterior layer of the femoral sheath an inch or so below Poupart's ligament, and internal to the femoral vein, which must be carefully guarded against injury, this suture may then be carried across the canal and through the lower border of Poupart's ligament. The needle at this stage should be unthreaded and withdrawn. The suture if now tied will obliterate the ring in a fairly satisfactory manner, but has caused some interference with the vein in certain cases. Other sutures may be introduced in a similar manner if necessary. The skin wound is afterwards closed, and firm pressure by means of a full antiseptic dressing obtained: a drainage tube is seldom necessary.

The parts will be healed in a few days, and the sutures from the superficial wound may be removed in a week or ten days. The recumbent position should be maintained for at least three weeks, and it is always my practice to apply a light form of truss after the operation when the patient begins to move about, as I am convinced that in the majority of cases a support of this kind is not only advisable but actually beneficial in the production of the continuance of the good results obtained by the herniotomy.

Some advocate that a sort of flap of the pectineal fascia, and possibly even some fibres of the pectineus muscle, be raised and sutured in position over the ring. I believe that this is but of little lasting service.

It has not been the scope of this paper to deal with strangulated femoral herniæ, though these are undoubtedly one variety of irreducible epiploceles.

